# What’s in SWAP? Abundance of the principal constituents in a soluble extract of *Schistosoma mansoni* revealed by shotgun proteomics

**DOI:** 10.1186/s13071-015-0943-x

**Published:** 2015-06-19

**Authors:** Leandro Xavier Neves, Ananda Lima Sanson, R. Alan Wilson, William Castro-Borges

**Affiliations:** Laboratório de Enzimologia e Proteômica, Instituto de Ciências Exatas e Biológicas, Universidade Federal de Ouro Preto, Ouro Preto, Minas Gerais Brazil; Centre for Immunology & Infection, Department of Biology, University of York, PO Box 373, York, YO10 5YW UK

**Keywords:** *Schistosoma mansoni*, Soluble proteome, Quantitative shotgun proteomics, Vaccine candidates

## Abstract

**Background:**

The soluble antigen preparation of adult schistosomes (SWAP) has often been used to probe host responses against these blood-dwelling parasites. Despite its long-established use there is limited knowledge about its composition. The information we provide here on the molecular composition of SWAP may contribute as a guide for a rational selection of antigenic targets.

**Methods:**

Label-free quantitative shotgun proteomics was employed to determine the composition and abundance of SWAP constituents. Briefly, paired adult *Schistosoma mansoni* worms were sonicated in PBS pH 7.2 and ultracentrifuged for recovery of the soluble supernatant. An aliquot was subjected to trypsin digestion. Resulting peptides were separated under ultra-high performance liquid chromatography and analysed using an orbitrap mass spectrometer. Spectral data were interrogated using SequestHT against an in-house *S. mansoni* database. Proteins were quantified by recording the mean area under curve obtained for up to three most intense detected peptides. Proteins within the 90^th^ percentile of the total SWAP mass were categorized according to their sub-cellular/tissue location.

**Results:**

In this work we expanded significantly the list of known SWAP constituents. Through application of stringent criteria, a total of 633 proteins were quantitatively identified. Only 18 proteins account to 50 % of the total SWAP mass as revealed by their cumulative abundance. Among them, none is predicted as a secreted molecule reinforcing the point that SWAP is dominated by cytosolic and cytoskeletal proteins. In contrast, only 3 % of the mass comprised proteins proposed to function at the host-parasite interfaces (tegument and gut), which could conceivably represent vulnerable targets of a protective immune response. Paradoxically, at least 11 SWAP proteins, comprising ~25 % of its mass, have been tested as vaccine candidates.

**Conclusions:**

Our data suggest that use of SWAP to probe host responses has greatest value for diagnostic purposes or assessing intensity of infection. However, the preparation is of limited utility as an antigen source for investigating host responses to proteins expressed at or secreted from worm-host interfaces. The data also pose the question as to why vaccination with SWAP, containing so many proposed vaccine candidates, has no additive or even synergistic effects on the induction of protection.

**Electronic supplementary material:**

The online version of this article (doi:10.1186/s13071-015-0943-x) contains supplementary material, which is available to authorized users.

## Background

SWAP (or SWA) is a soluble adult worm antigen preparation universally used by immunologists to probe host responses to schistosome infections. It is essentially a Tris-HCl or PBS-based extract of mixed male and female worms variously prepared by homogenization, sonication or freeze/thaw (or a combination of these) followed by a high-speed centrifugation to remove particulate material. It yields a solution typically containing 3-5 mg protein/mL which can be stabilized by adding protease inhibitor cocktail for ELISA and Western blotting or used without inhibitors for cell-stimulation assays. The first report on its use appears to be from 1977 [1] and 38 years later it is still a standard preparation. In one recent publication SWAP was used to investigate human anti-fecundity immunity to *Schistosoma haematobium* using antibody isotype ELISAs as the assay of reactivity [2]. In a second study, a version of SWAP supplemented by proteins soluble in chaotropic agents and mild detergents provided the material for an immunoproteomic analysis of human antibody responses, using 2D western blotting to select reactive constituents [3].

In spite of this long-established usage very little is known about its precise composition. The first proteomic study of *S. mansoni*, using 2D-gel technology and peptide-mass fingerprinting, provided a comparison of soluble preparations from four different life-cycle stages, among them adult worms [4]. The 40 most abundant spots from 2D gel comprised glycolytic enzymes (e.g enolase, aldolase, GAPDH, TPI), cytoskeletal proteins (actin, myosin light chain, tropomyosin), chaperones (Hsp-70, 14-3-3), stress response proteins (superoxide dismutase, GST-28) and calcium-binding proteins (calponin and EF-hand Sm21.7). However, no information was provided on relative abundance of the components. Peptide mass fingerprinting was superseded more than a decade ago by peptide fragmentation as a more accurate means of protein identification. Whilst gel technology is still useful in some applications, for compositional studies quantitative shotgun proteomics is now the method of choice [5]. Furthermore, the advances in mass spectrometer design have increased the sensitivity of peptide detection by orders of magnitude [6, 7].

Motivated by a feeling that immunologists really ought to know exactly what they are probing their cell or antibody responses with when they use SWAP, we describe here an analysis of its composition using state-of-the-art instrumentation in conjunction with software for protein identification and quantitation. We have shown that at least 80 % of the identified molecules are primarily of intracellular origin whereas only 3 % represent signature proteins derived from the tegument and gastrodermis, the major host-parasite interfaces. The information we provide on the composition and abundance of SWAP constituents should better enable immunologists to appreciate exactly what antigenic reactivities they are monitoring.

## Methods

### Ethics statement

The protocol for maintenance of the *S. mansoni* life cycle was reviewed and approved by the local ethics committee on animal experimentation, Comissão de Ética no Uso de Animais (CEUA), Universidade Federal de Ouro Preto (UFOP), and received the protocol no. 2011/55.

### SWAP or (SWA) – soluble worm antigen preparation

Approximately 100 adult paired worms were sonicated in 1 mL PBS pH 7.2 containing 1x Protease Inhibitor Cocktail (Sigma Aldrich, St. Louis, USA). The homogenate was clarified by centrifugation at 40,000 *x g*, for 2.5 h, at 4 °C followed by recovery of the supernatant containing soluble antigens. The protein content was determined using PIERCE™ BCA Protein Assay Kit (Thermo Scientific, Rockford, USA).

### In solution digestion and mass spectrometry analysis

Proteins present in a 50 μg aliquot of SWAP were first reduced using 2 mM dithiothreitol (Sigma Aldrich) at 56 °C, for 15 min, in 100 mM ammonium bicarbonate. After cooling at room temperature, proteins were then alkylated in 4.5 mM iodoacetamide (Sigma Aldrich) for 15 min in the dark. The sample was then diluted 2.5 times using ultra-pure water and the ammonium bicarbonate concentration adjusted to 100 mM. Protein digestion was carried out at 37 °C for 16 h using Sequencing Grade Modified Trypsin (Promega, Madison, USA) at a protein to enzyme ratio of 25:1. Trypsinolysis was terminated by sample acidification with 4 % (*v/v*) ultra-pure glacial acetic acid (J.T. Baker, Center Valley, USA). Peptides were cleaned up by solid phase extraction using a Strata C_18_-E cartridge (55 μm, Phenomenex, Macclesfield, UK).

### LC-MS/MS analyses

Five μg of the peptide preparation were loaded onto a UltiMate® 3000 UHPLC system (Thermo Scientific, Bremen, Germany) equipped with an Acclaim PepMap100 C_18_ Nano-Trap Column (75 μm i.d. × 2 cm, 3 μm, 100 Å; Thermo Scientific) in line with an Acclaim PepMap100 C_18_ RSLC (75 μm i.d. × 15 cm, 2 μm, 100 Å; Thermo Scientific) capillary column. Trapped peptides were washed for 3 min in 2 % (*v/v*) acetonitrile/0.05 % (*v/v*) trifluoracetic acid, at a flow rate of 5 μL/min, before switching the flow to the capillary column. Peptide separation was carried out at 40 °C under a multi-step gradient using a combination of solvents A (0.1 % (*v/v*) formic acid), and B (80 % (*v/v*) acetonitrile/0.1 % (*v/v*) formic acid). The gradient started from 4 % solvent B, increased to 30 % over 180 min and reached 55 % at 240 min. This was followed by a sharp ramp to 90 % solvent B over 10 min, maintenance at 90 % for an additional 10 min and finally a drop to 4 % over 20 min to restore the initial condition. All UHPLC solvents were purchased from J.T. Baker®.

A nano UHPLC system interfaced with a Q-Exactive™ instrument (Thermo Scientific) allowed the mass spectrometric analysis of eluting peptides. A Nanospray Flex Ion Source (Thermo Scientific) fitted with a stainless steel nano-bore emitter needle (150 μm o.d. × 30 μm i.d., Proxeon, Thermo Scientific) was operated at 1.9 kV, in positive mode and capillary temperature set to 250 °C. Survey scans were acquired at a resolution of 70,000 with maximum injection time of 100 ms and target value of 3 × 10^6^ ions. Up to 12 most abundant isotope patterns scanned in the range of 300-2000 *m/z*, exhibiting charge state ≥2 were isolated over a window of 2 *m/z* before fragmentation via higher-energy collisional dissociation (HCD) with normalized collision energy of 30 V. MS/MS spectra were acquired at a 17,500 resolution with maximum injection time of 150 ms and target value of 2 × 10^5^ ions. Dynamic exclusion of ions was set to 60 s.

### Data processing

Mass spectral data were submitted to database search using Proteome Discoverer software v.1.4 (Thermo Scientific). Our workflow included the SequestHT search engine plus Event Detector and Precursor Ions Area Detector for label-free quantification. Searching parameters included: 1) enzyme, trypsin/P; 2) maximum missed cleavage sites = 2; 3) carbamidomethyl (C); 4) methionine oxidation and N-terminal acetylation as dynamic modifications; 5) mass tolerance of 10 ppm for parental ions and 0.1 Da for MS/MS. Spectra were searched against a local *Schistosoma mansoni* database (10,773 sequences; 5,135,674 residues). Only the protein identities that matched to at least one high confidence peptide were considered.

### Protein categorization and bioinformatic analyses

Proteins were categorized by sub-cellular or tissue location using data from the *S. mansoni* database, Uniprot, and Blast searches. Signature proteins, here defined as known constituents of the soluble, gut-secreted and tegument proteomes, were listed based on published literature [4, 8–11]. Signal peptides were predicted using SignalP v3.0.

### Statistical analysis

Arbitrary values related to individual protein abundance were tested for normal distribution using the Shapiro-Wilk test by the GraphPad Prism version 5.00 for windows, GraphPad software, San Diego, California, USA.

## Results

### Shotgun analysis revealed a large number of constituents in SWAP

A total of 60,571 peptides, selected by the Q-Exactive for fragmentation, yielded approximately 1000 identities when the latest version of the *S. mansoni* gene predictions was searched using Proteome Discoverer. We then used the area under the curve of each fragmented precursor ion, recorded by the software, as the measure of protein abundance. To ensure we did not eliminate low mass proteins with few possible tryptic peptides (e.g cyclophilin and ubiquitin) we included identities based on a single peptide provided that the MS/MS spectrum was of sufficient quality to guarantee a high confidence score. This reduced the data for analysis to 633 proteins at a false discovery rate of 1 %.

### Abundance of SWAP constituents is not evenly distributed

**Fig 1:**
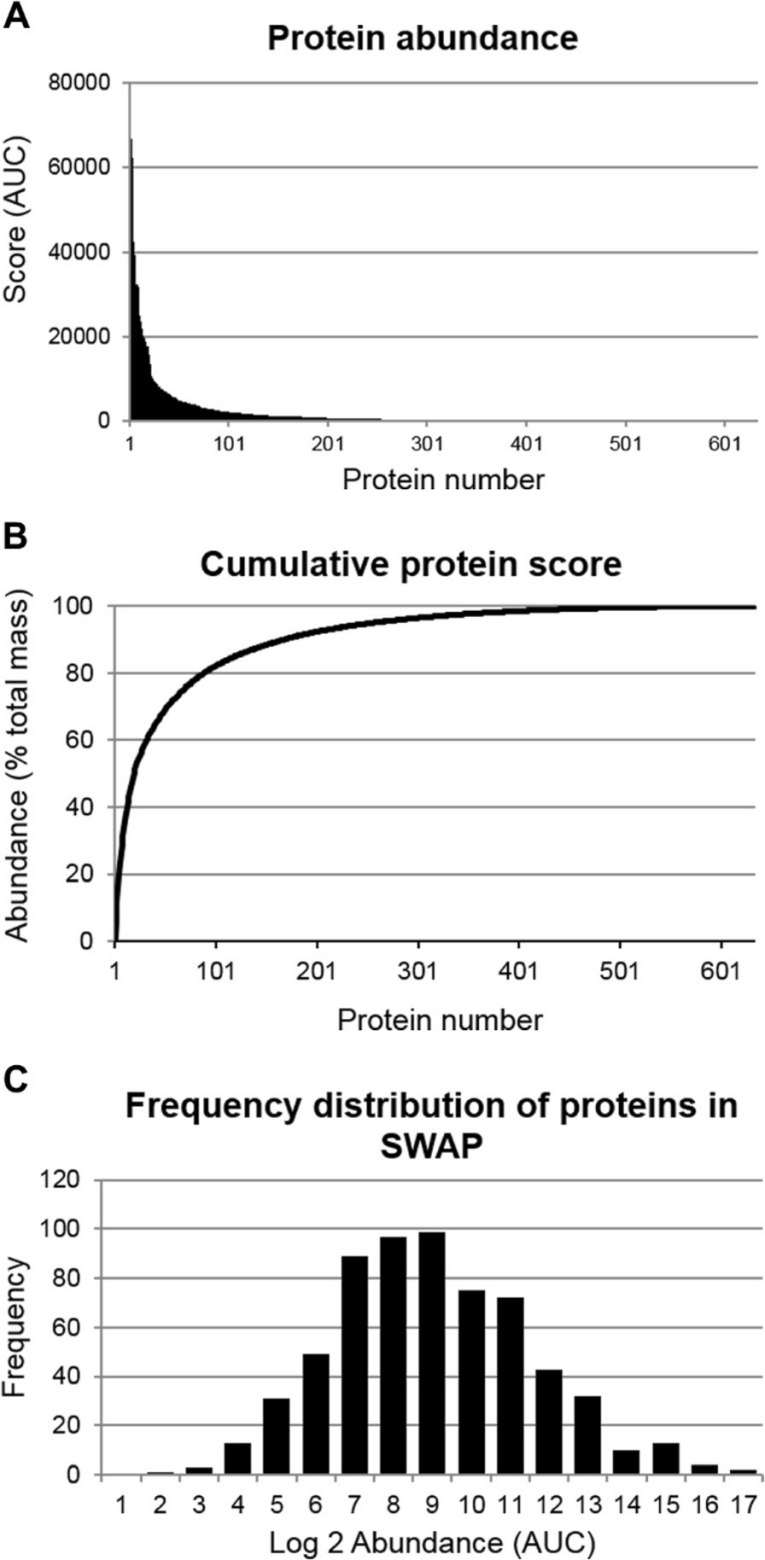
Protein distribution in SWAP. **a** Protein abundance of SWAP determined using the absolute Area Under Curve (AUC) values recorded for each of the 633 proteins identified. Note that a very small number of proteins contributed to the most prominent MS peaks detected. **b** Protein abundance in SWAP represented as a cumulative frequency plot revealed that only 18 constituents contributed to 50 % of the total mass. **c** Frequency distribution plotted after Log_2_ transformation of the calculated AUC for each protein. Although the distribution superficially appears Gaussian, a Shapiro-Wilk test revealed that it is not (*p* < 0.05), high abundance proteins being over-represented

An intensity plot of the recorded area score for each identified protein shows a markedly skewed distribution with a relatively small number of proteins being highly abundant and many present in only trace amounts (Fig. 1a). The dynamic range of modern proteomic analysis was revealed by the highest (78,766) and lowest (3.6) values in the 633 protein series, which differed by more than four orders of magnitude. The proteins were arranged in descending order by abundance to record the cumulative total area, and the percentage contribution of each determined (Fig. 1b). Twenty percent of the total SWAP mass was contributed by only five proteins, namely enolase, aldolase, actin, glyceraldehyde 3-phosphate dehydrogenase and fatty acid-binding protein (Table 1). Fifty per cent of the total mass was represented by only 18 proteins and a further 149 made up 90 % of the total. The remaining 10 % was accounted for by 467 proteins each present in very small amounts. We chose to focus subsequent analyses on the 167 proteins that comprise the bulk of the mass up to the 90^th^ percentile. Proteomic analyses of other systems have revealed that the log concentration of protein abundance displays a normal distribution. Our SWAP preparation (Fig. 1c) appears visually skewed towards the more abundant components so we tested its normality using Shapiro-Wilk test proving a non-Gaussian distribution (*p* = 0.016).

**Table 1:** Top 18 most abundant proteins representing half of the total mass in SWAP

Gene DB Accession	Description	Class	# of Peptides	% Coverage	PD Area^a^	MW [kDa]	% total SWAP	*Homo sapiens* Accession	% Identity	% Cons.	Signal peptide
Smp_024110.1	Enolase	EM	12	33,4	78,766	47,0	6,1	NP_001419.1	75	86	no
Smp_042160.1	Aldolase	EM	11	27,2	66,915	35,4	5,2	NP_005156.1	69	80	no
Smp_161930.1	Actin	CY	7	30,3	62,296	35,5	4,8	gb|AAP37280.1	67	71	no
Smp_056970.1	Glyceraldehyde 3 P dehydrogenase	EM	6	25,7	48,106	36,4	3,7	NP_002037.2	73	82	no
Smp_095360.1	Fatty acid-binding protein, *Sm14*	ILT	4	39,9	42,434	14,8	3,3	gb|AAB87141.1	47	61	no
Smp_054160.1	Glutathione S-transferase, GST 28	SR	7	42,7	39,378	23,8	3,1	AAA58623.1	30	48	no
Smp_106930.1	Heat shock 70 kDa protein	CH	17	29,7	32,378	69,8	2,5	NP_006588.1	84	92	no
Smp_050390.1	Aldehyde dehydrogenase	EM	11	31,4	32,212	53,7	2,5	gb|AAH01619.1	57	72	no
Smp_009760.1	*14-3-3 protein zeta/delta*	CH	6	24,6	31,822	28,4	2,5	NP_003397.1	64	74	no
Smp_040130.1	Cyclophilin	CH	1	8,70	30,114	17,6	2,3	AAH05982.1	67	73	no
Smp_034840.1	14-3-3 protein epsilon	CH	11	48,0	24,916	26,0	1,9	NP_006752.1	65	82	no
Smp_086330.1	*Calponin*	CB	7	44,2	23,786	21,2	1,8	XP_006722711.1	47	64	no
Smp_183710.1	Actin	CY	7	27,7	21,927	41,7	1,7	NP_001092.1	96	99	no
Smp_049550.1	78 kDa glucose regulated protein	CH	15	25,0	20,302	71,2	1,6	NP_005338.1	75	87	yes
Smp_045200.1	Tegument-allergen-like protein *Sm22.6*	CB	5	25,8	19,594	22,6	1,5	pdb|2PMY|A	29	50	no
Smp_008070.1	Thioredoxin	SR	7	71,7	19,167	11,9	1,5	NP_003320.2	47	61	no
Smp_086530.1	Tegument-allergen-like protein *Sm20.8*	CB	7	47,0	18,922	20,8	1,5	BAD97069.1	22	49	no
Smp_096760.1	Phosphoglycerate mutase	EM	10	50,0	17,642	28,4	1,4	NP_000281.2	57	71	no
		Means	8,4	35,2	35,038	33,7	2,7		60	72	

Annotations in italics were added by the authors

*EM* Energy metabolism, *CY* Cytoskeletal, *ILT* Intracellular lipid transport, *SR* Stress response, *CH* Chaperone, *CB* Calcium binding

^a^The Proteome Discoverer (PD) units of area under curve were divided by 10^6^ for ease of manipulation

### SWAP constituents are heavily biased towards cytosol and cytoskeleton

**Fig 2:**
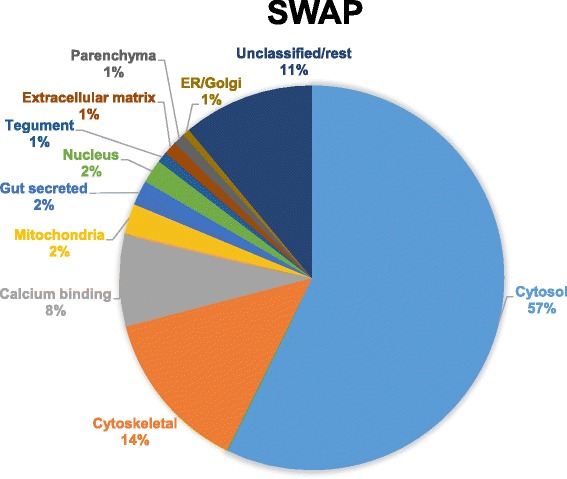
Sub-cellular or tissue location of SWAP constituents. Proteins within the 90^th^ percentile were categorized according to published literature, Uniprot and Blast searches. Soluble cytosolic and cytoskeletal proteins contribute to over 70 % of total abundance in SWAP. Conversely, proteins proposed to function at host-parasite interfaces (e.g gut secreted and tegument surface) account for only 3 % of the total mass

We first searched the total dataset for matches to signature proteins identified by our previous proteomic investigations, color-coding the observed similarities. Sixteen out of 17 proteins identified by Curwen *et al*. 2004 [4] were equally prominent in our cytosolic fraction, many of them occupying the top positions in both studies. Approximately half the proteins identified as constituents of the worm vomitus were detected; these were almost exclusively the hydrolytic enzymes not the transporter proteins. Only seven out of 61 verified tegument surface proteins were present in SWAP, with three, Sm200, Sm29 and an annexin within the 90^th^ percentile. We then grouped each one of the 167 top proteins by sub-cellular or tissue location, by manual searching of several databases. Ten putative proteins were unusable or they were hypothetical, not yielding a meaningful annotation after BLAST searching of NCBInr, and were transferred to the residual unclassified category. These were approximately balanced by adding the known gut and tegument proteins in the bottom 467 identities. The color-coded proteins were then sorted by function/tissue category and the total mass contributed by each group illustrated in a pie-chart (Fig. 2).

The largest category by far (~60 %) comprises intracellular proteins from the cytosol. They function principally in glycolysis, protein translation, protein folding and the response to stress, all classical housekeeping activities. The second largest category (14 %) comprises proteins of the cytoskeleton and associated proteins, many of which will be present in the musculature. We classified calcium-binding proteins as a separate category (8 %) since they are found in many cellular locations and tissues like the tegument. Although mitochondria and nuclei are abundant cellular constituents, proteins originating therein comprised only 2.5 and 2 % of the total, respectively. The few mitochondrial proteins appear to come from the matrix, likely released upon sonication (Additional file 1). The total mass contribution of tegument and gut proteins amounts to only 3 %. Further small contributions of protein are made by extracellular matrix, the secretory pathway and glycogen metabolism, this last representing parenchymal tissue.

### A small number of proteins comprise half the mass

A detailed consideration of the top 18 proteins comprising half the total mass revealed that all were intracellular with only one (Smp_049550, 78 kDa glucose regulated protein; Table 1) possessing a signal peptide (*p* = 0.999). However, this protein is a Hsp-70 family member involved in protein folding and localized to the lumen of the endoplasmic reticulum. The remaining 17 proteins are all located in the cytosol rather than in an organelle and so unlikely to be N- or O- glycosylated for export (Table 1). When grouped by cellular function the five enzymes involved in energy metabolism (Table 1, EM) accounted for 39 %, and the four chaperones (CH) for 22 %. The two cytoskeletal proteins (CY) comprise 13 %, the three calcium-binding proteins 10 % and the two stress-response proteins (SR) 9 %. Fatty acid-binding protein, involved in intracellular lipid transport (ILT), made up the final 7 %. The abundance of all these proteins, apart from cyclophilin, is confirmed by the large number of diagnostic peptides (range 4-17, mean 8.4) observed for each, amounting to a mean of 36.2 % sequence coverage (range 9-72 %). All the top 18 identities are medium-sized proteins (range 12-71 kDa, mean 33.7 kDa). Fifteen out of 18 proteins have close homology in the human host (mean 66 %), the three exceptions being the EF-hand calcium-binding proteins Sm22.6 and Sm20.8 and glutathione S-transferase 28 (mean 27 %). It is notable that, at least three proteins in the first group and two in the second have been proposed as vaccine candidates.

## Discussion

In this study we have provided the first inventory of SWAP constituents using a shotgun-based proteomic approach. The applied methodology combines high-resolution liquid chromatography for peptide separation coupled to accurate *m/z* detection of eluting molecules using a mass spectrometer. Compared to the classic 2D gel technique, shotgun analysis allows for detection of proteins with a wide dynamic range, differing in abundance by several orders of magnitude. In addition to protein identification, the shotgun strategy is also capable of estimating protein abundance through peak area detection of a precursor ion, using either tag-modified peptides [12], SILAC [13] or label-free methods [14, 15]. The last alternative requires minimal sample handling and can be applied to any detectable peptide, excluding the need for chemical tagging at specific amino acid residues. Here we have applied a stringent criterion for peptide selection, and hence protein identification, to gain insight into the relative abundance of the major constituents of the SWAP preparation. This approach increased significantly the repertoire of soluble worm protein constituents first described in 2004 using 2D gel electrophoresis [4].

Protein categorization revealed a preparation largely containing intracellular constituents that accounted for 97 % of the identities. In the context of schistosome biology, this large fraction of proteins will only trigger immune effector mechanisms of the vertebrate host when parasite larvae, adult worms or eggs are damaged or die, releasing their cytosol into the host bloodstream or tissues. By extension, the protein constituents of SWAP are unlikely to be targets of a protective immune response because they are largely confined within the cells of live parasites. In stark contrast, proteins expressed at the host-parasite interfaces (namely the tegument and alimentary tract) are for the most part scarce or undetectable in SWAP accounting for only approximately 3 % of the total mass. In the case of proposed tegument location, the only proteins detected were putative constituents of the membranocalyx namely Sm200, an annexin, CD59 and Sm29 [10, 11]. Concerning the last of these, its recorded relative abundance was too small to be included in the 90^th^ percentile of SWAP constituents. Annexin aside, the other three proteins Sm200, CD59 and Sm29 are known to be GPI-anchored [11], and their presence in SWAP could be explained by physical extraction of such membrane proteins during sample homogenization. Based on the aforementioned percentages it is clear that when SWAP is used to probe host cellular or antibody responses, the fraction of these attributable to surface-exposed or secreted proteins is negligible. It is noteworthy to mention we only considered secretion/excretion of proteins by means of the classical secretory pathway. Previous studies performed *in vitro* reported the production of vesicles by schistosomes [16, 17] and exosome-like particles being formed in the tegument of other parasitic helminths (*Echinostoma caproni* and *Fasciola hepatica*) [18]. However, compositional analysis of the *S. mansoni* culture’s supernant and the lipidome of the host’s blood plasma did not indicate the release of phospholipids known to be enriched at the parasite’s tegument surface [19]. Identification of such lipids would argue in favour of genuinely secreted exosomes. Thus, to our understanding prediction of signal peptide remains the most convenient approach to anticipate protein secretion by *S. mansoni*.

We tacitly acknowledged this limitation of SWAP in our study of rhesus macaque self-cure responses [20] where we developed two specific antigen fractions, SASP (Stimulated Adult Worm Secreted Preparation) and TSP (Tegument Surface Preparation), to investigate reactivity to gut and tegument constituents, respectively, by Western blotting. At least two major conclusions could be drawn from these macaque experiments. First, only low burden animals exhibited high antibody titers against those antigens. A major fraction of the reactive spots could not be easily visualized using high sensitivity staining methods of the corresponding replica gels, suggesting that likely protective antibodies are targeting minor components of the preparations. Second, particularly for the tegument enriched preparation, the complexity of serum reactivity is inversely proportional to final worm burden. It follows that any similar studies wishing to investigate host responses to proteins expressed at or secreted from interfaces will have limited utility if SWAP is used as the only antigen source.

SWAP preparations have been used for vaccination experiments in past decades with variable results [21, 22]. The most consistent results were obtained in an extensive series of experiments where 1 mg of SWAP was administered intradermally to mice along with 5 × 10^6^ units of BCG [23, 24]. The BCG alone elicited no protection whilst SWAP alone produced a small non-significant effect [25]. Paramyosin, a muscle protein was the major immunogen detected by antibodies from vaccinated animals although paradoxically this group of researchers concluded that the 40-50 % protection achieved was based on T cell-mediated mechanisms involving interferon gamma production by T cells and activation of macrophages, not humoral responses [25]. Paramyosin is #46 in our SWAP preparation by abundance, equating to 0.4 % of the total protein or 4.4 μg/mg. This is exactly in the range of affinity-purified paramyosin subsequently used to achieve ~40 % protection in mice with BCG as adjuvant [26]. In this same publication the authors note that the SWAP-depleted of paramyosin still possessed protective properties. This leads us directly to the observation that many of the most abundant constituents of our SWAP from *S. mansoni* (or *S. japonicum*) have been administered to mice to induce protective immunity.

A search of the literature reveals that these candidates include aldolase (#2), GAPDH (#4), fatty acid-binding protein (#5), GST-28 (#6), 14-3-3 (#9), calponin (#12), Sm22.6 (#15) and Sm20.8 (#17) [27–34] with levels of protection ranging from 30 to 50 %. The respective abundance of these proteins in 1 mg of our SWAP preparation is 52, 37, 33, 31, 25, 18, 15 and 15 μg, collectively amounting to 226 μg. If we add in other proposed candidates such as Cu/Zn superoxide dismutase [35] (#21, 15.3 μg), myosin heavy chain [36] (#32, 6 μg) and protein disulphide isomerase [37] (#34, 5.8 μg) it is clear that at least a quarter of SWAP by mass comprises putative vaccine candidates. However, there is clearly no additive effect when all these candidates are administered together as the SWAP cocktail. Indeed, this is an odd and unexplained feature of the protection induced in mice by what are almost entirely cytosolic and cytoskeletal (i.e. internal) candidates. It seems each must be triggering the same mechanism to approximately the same degree but the outcome always hits a ceiling of approximately 40 % protection whether the antigen is given alone or *en masse* via SWAP. We intend to address this conundrum in a separate publication.

The choice of SWAP to probe host responses is pragmatic because it is easy to prepare, the yields of soluble protein are high and due to the intrinsic immunogenicity of its major constituents, it virtually guarantees a strong response in assays. However, our data suggest that this last point is a trap for the experimenter. The principal components we have identified have been termed cryptic antigens [2] because they are only released when the adult worm dies. Proteomic analysis has revealed a similar mixture of soluble proteins in the eggs that are deposited and die in the tissues [4, 38] so that the host will be continuously restimulated by major SWAP constituents throughout the course of an infection. It is worth to bear in mind that SWAP may contain other classes of antigens not particularly dealt with in this shotgun approach. Anyhow, lipids and glycolipids are unlikely to be enriched in SWAP as no organic solvents (e.g methanol-chloroform) are used during its preparation. In addition, we cannot rule out the possibility that identification of glycoproteins has been made in this study. This can be achieved by identification and quantitation of non-glycosylated tryptic peptides.

Recently, the rate of evolution of schistosome proteins has been investigated using the dN/dS ratio of nucleotide substitutions as the criterion of selection pressure, in the three major schistosome species that infect humans [39]. The study revealed that genes encoding exposed micro-exon gene (MEG) proteins and some venom allergen-like (VAL) proteins had the highest ratios, compared to genes encoding internal proteins and gastrodermal secretions. This applied even to those proteins at the tegument surface, apart from two surface vaccine candidates, Sm29 and the extrinsic portion of tetraspanin TSP-2, which also had high dN/dS ratios. These data indicate that there is a definitive tendency in the Genus *Schistosoma* for truly exposed antigens to diversify due to selection pressure, whereas the cryptic antigens vary much less. A strong immune response is mounted against the cryptic antigens when they are encountered, but cannot provide effective protection. Thus, survival of a schistosome population, and adaptation to new hosts, requires an evolutionary response that modifies exposed epitopes to extend longevity and facilitate worm reproduction. Paradoxically, while estimation of the dN/dS ratio provides an indicator of diversification, by extension it also reveals that the existing parasite population has been able to accommodate the hostile response. Our compositional analysis of SWAP, in which the highly modified MEGs and VALs proteins are barely detectable, suggests that its use to probe host responses to find vaccine candidates is almost certain to point the researcher in the wrong direction. Furthermore, where major SWAP components (or SWAP itself) have been tested in vaccination experiments the ~40 % protection observed has some rational explanation other than specific acquired immunity.

## Conclusion

In this work we conducted a quantitative shotgun proteomic analysis of SWAP greatly expanding the list of its constituents. Our data have shown that at least 80 % of the identified molecules are primarily of intracellular origin whereas only 3 % represent signature proteins derived from the tegument and gastrodermis, the major host-parasite interfaces. This finding should allow immunologists to appreciate the dominant targets of the host cellular and antibody responses they are monitoring when SWAP is used as an antigenic preparation. This study finally poses the question as to why vaccination with SWAP, containing so many proposed vaccine candidates, has not shown additive or even synergistic effects on the induction of protection.

## Additional file

Additional file 1:
**Protein/peptide mass spectrometry data and derived parameters.**
